# Prevalence, Antimicrobial Resistance, and Molecular Characterization of Vancomycin-Resistant Enterococci in a North Indian Tertiary Care Hospital

**DOI:** 10.7759/cureus.102696

**Published:** 2026-01-31

**Authors:** Neeraj Kumar Verma, Manodeep Sen, Nikhil Raj, Anupam Das, Jyotsna Agarwal

**Affiliations:** 1 Microbiology, Dr. Ram Manohar Lohia Institute of Medical Sciences, Lucknow, IND

**Keywords:** enterococcus faecium, gram-positive bacteria, nosocomial infections, vancomycin, vancomycin-resistant enterococci

## Abstract

Background

Vancomycin-resistant enterococci (VRE) are important nosocomial pathogens with limited treatment options and increasing prevalence in India. This study aimed to determine the prevalence of VRE among hospitalized adults, evaluate their antimicrobial resistance patterns, and characterize genetic determinants of vancomycin resistance.

Methods

A cross-sectional study was conducted from April 2023 to September 2024. *Enterococcus *spp. isolated from adult inpatients were identified using standard methods and matrix-assisted laser desorption/ionization time-of-flight mass spectrometry (MALDI-TOF MS). Antimicrobial susceptibility testing was performed by Kirby-Bauer disc diffusion and Epsilometer strip minimum inhibitory concentration (E-strip MIC) testing. A subset of isolates underwent multiplex real-time polymerase chain reaction (PCR) for vanA and vanB gene detection.

Results

Of 3,176 *Enterococcus *isolates, 234 (7.36%) were VRE. Urine samples accounted for 210/2,684 (7.8%) and blood cultures for 12/210 (5.7%) of VRE isolates. Overall, *Enterococcus* faecalis (*E. faecalis)* (1,960; 61.7%) predominated, while *Enterococcus faecium* (*E. faecium)* (1,216; 38.3%) showed higher resistance. Among VRE, *E. faecalis* contributed 145/234 (61.9%) and *E. faecium* 89/234 (38.0%). All VRE were resistant to vancomycin (MIC ≥32 µg/mL). Linezolid was effective against all isolates (234/234; 100%). Molecular analysis of 20 representative isolates confirmed the presence of vanA in all (100%), with no vanB detected.

Conclusion

A 7.36% prevalence of VRE was observed, predominantly in urinary and bloodstream infections. Exclusive detection of vanA indicates clonal dissemination of high-level resistance. Routine species-level identification, antimicrobial stewardship, and molecular surveillance are critical to limiting VRE spread in tertiary care settings.

## Introduction

Enterococci, primarily *Enterococcus faecalis* (*E. faecalis*) and *Enterococcus faecium* (*E. faecium*), are facultative anaerobic, Gram-positive cocci that form part of the normal flora of the gastrointestinal tract. However, over the past few decades, these organisms have emerged as significant opportunistic pathogens responsible for a wide range of healthcare-associated infections (HAIs), including urinary tract infections (UTIs), bloodstream infections (BSIs), endocarditis, intra-abdominal infections, and wound infections, particularly in hospitalized or immunocompromised individuals [1]. Among the enterococcal species, *E. faecium* has gained increasing clinical relevance due to its heightened resistance to multiple classes of antibiotics [2]. Of particular concern is its resistance to vancomycin, a glycopeptide antibiotic traditionally considered a last-resort agent for multidrug-resistant Gram-positive infections. Vancomycin-resistant enterococci (VRE), first reported in the late 1980s, have since been identified globally, with varying prevalence across regions and hospital settings [3]. India has seen a progressive rise in antimicrobial resistance, including VRE, driven by unregulated antibiotic use, prolonged hospitalizations, high patient loads in tertiary care centers, and inadequate infection control practices [4]. However, comprehensive data on the burden of VRE infections, particularly from the northern region of India, remains sparse [5]. Accurate identification and surveillance of VRE are essential, as infections caused by these organisms are associated with increased morbidity, mortality, prolonged hospital stays, and higher healthcare costs [6]. Furthermore, therapeutic options for VRE are limited and often include expensive or toxic alternatives such as linezolid, daptomycin, or tigecycline [7]. This study aimed to determine the prevalence of VRE among hospitalized adults, evaluate their antimicrobial resistance patterns, and characterize genetic determinants of vancomycin resistance.

## Materials and methods

This cross-sectional observational study was conducted prospectively in the Department of Microbiology, Dr. Ram Manohar Lohia Institute of Medical Sciences (Dr. RMLIMS), Lucknow, Uttar Pradesh, India, over an 18-month period from April 2023 to September 2024. Consecutive sampling was employed, wherein all eligible *Enterococcus *isolates received during the study period were included. A total of 3,176 clinical *Enterococcus *isolates were obtained, of which 234 (7.36%) were identified as VRE and constituted the final study sample.

A wide variety of clinical specimens, including urine, blood, pus, wound swabs, sputum, and body fluids (cerebrospinal, pleural, peritoneal, and synovial), were collected using standard sterile techniques, and all sample collection, transport, and processing were performed in accordance with CDC guidelines [8]. *Enterococcus *isolates were initially identified using standard microbiological methods, with biochemical characterization employed to differentiate *E. faecalis* and *E. faecium* [9]. Final species-level confirmation was performed using matrix-assisted laser desorption/ionization time-of-flight mass spectrometry (MALDI-TOF MS; VITEK® MS, bioMérieux, Marcy-l’Étoile, France). Identification was considered reliable when the confidence value provided by the system was ≥99.9%, in accordance with the manufacturer’s recommendations. Antimicrobial susceptibility testing (AST) was carried out by the Kirby-Bauer disc diffusion method on Mueller-Hinton agar using commercial antibiotic discs (HiMedia, Thane, India), and results were interpreted according to CLSI M100, 32nd edition breakpoints [10].

Genomic DNA was extracted from pure cultures using the HiPurA® Bacterial Genomic DNA Purification Kit (HiMedia) following the manufacturer's instructions. A real-time multiplex polymerase chain reaction (PCR) was performed using the Hi-PCR® VRE Multiplex Probe PCR Kit (HiMedia), targeting vanA, vanB, and the *Enterococcus *genus-specific 16S rRNA gene. PCR was carried out in a calibrated real-time thermocycler under recommended cycling conditions. Positive, negative, and internal controls were included in each run. Results were interpreted based on Ct values and amplification curves.

Statistical analysis 

Categorical variables, including age groups, gender, sample type, species distribution, and antibiotic susceptibility patterns, were summarized as frequencies and percentages. Statistical analysis was performed using GraphPad Prism software (La Jolla, CA, USA). The Chi-square (χ²) test was used to assess associations between categorical variables after verifying test assumptions. Fisher’s exact test was applied when expected cell counts were <5. A p-value <0.05 was considered statistically significant.

## Results

**Figure 1:**
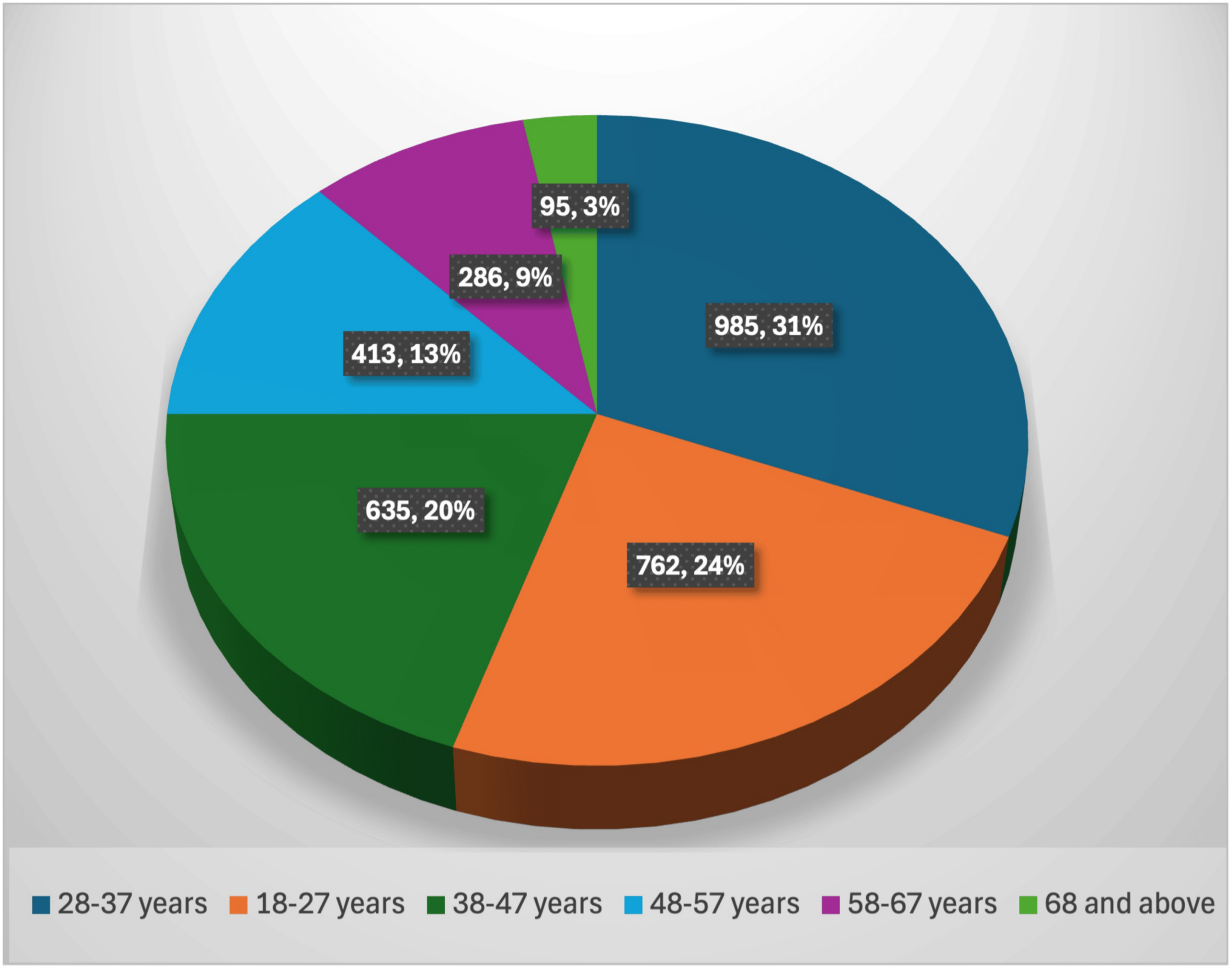
Age-wise distribution of Enterococcus infections among hospitalized adults

A total of 3,176 clinical *Enterococcus *isolates were processed during the study period, of which 234 (7.36%) were identified as VRE. Demographic analysis of all *Enterococcus *isolates (n = 3,176) showed that the highest proportion of isolates was observed in the 28-37-year age group (982/3,176; 30.95%), followed by the 18-27-year age group (771/3,176; 24.28%), with a declining trend in frequency with increasing age. This age-wise distribution was statistically significant (p < 0.001). Gender-wise distribution among all *Enterococcus *isolates demonstrated a female predominance (2,130/3,176; 67%) compared to males (1,046/3,176; 33%), which was also statistically significant (p < 0.001). The age-wise distribution of *Enterococcus *infections is illustrated in Figure 1. 

VRE isolates were recovered from a variety of clinical specimens, with the highest frequency observed in urine samples (210/2,684; 7.8%), followed by blood (12/210; 5.7%), while no VRE were detected in bronchoalveolar lavage or sputum samples (0/19; 0%). Statistical analysis showed no significant difference in VRE prevalence between urine and blood samples (p = 0.224). Species distribution among all *Enterococcus *isolates revealed that *E. faecalis* accounted for 1,960/3,176 (61.7%) and *E. faecium* 1,216/3,176 (38.3%). *E. faecalis* was most frequently isolated from urine, whereas* E. faecium* predominated in blood, a difference that was statistically significant (p < 0.001). Among the 234 VRE isolates, *E. faecalis* represented 145 of 234 (61.97%) and *E. faecium* 89 of 234 (38.03%) samples, with no significant deviation from the overall species proportions (p = 0.968). The detailed distribution of VRE by sample type and species is summarized in Table 1.

**Table 1:** Distribution of vancomycin-resistant enterococci (VRE) by sample type

Sample Type	Total Isolates(%)	VRE Isolates	Frequency
Urine	2684	210	7.8%
Blood	210	12	5.7%
Pus	175	9	5.1%
Body fluids	88	3	3.4%
Bronchoalveolar lavage, sputum	19	0	0%

**Figure 2:**
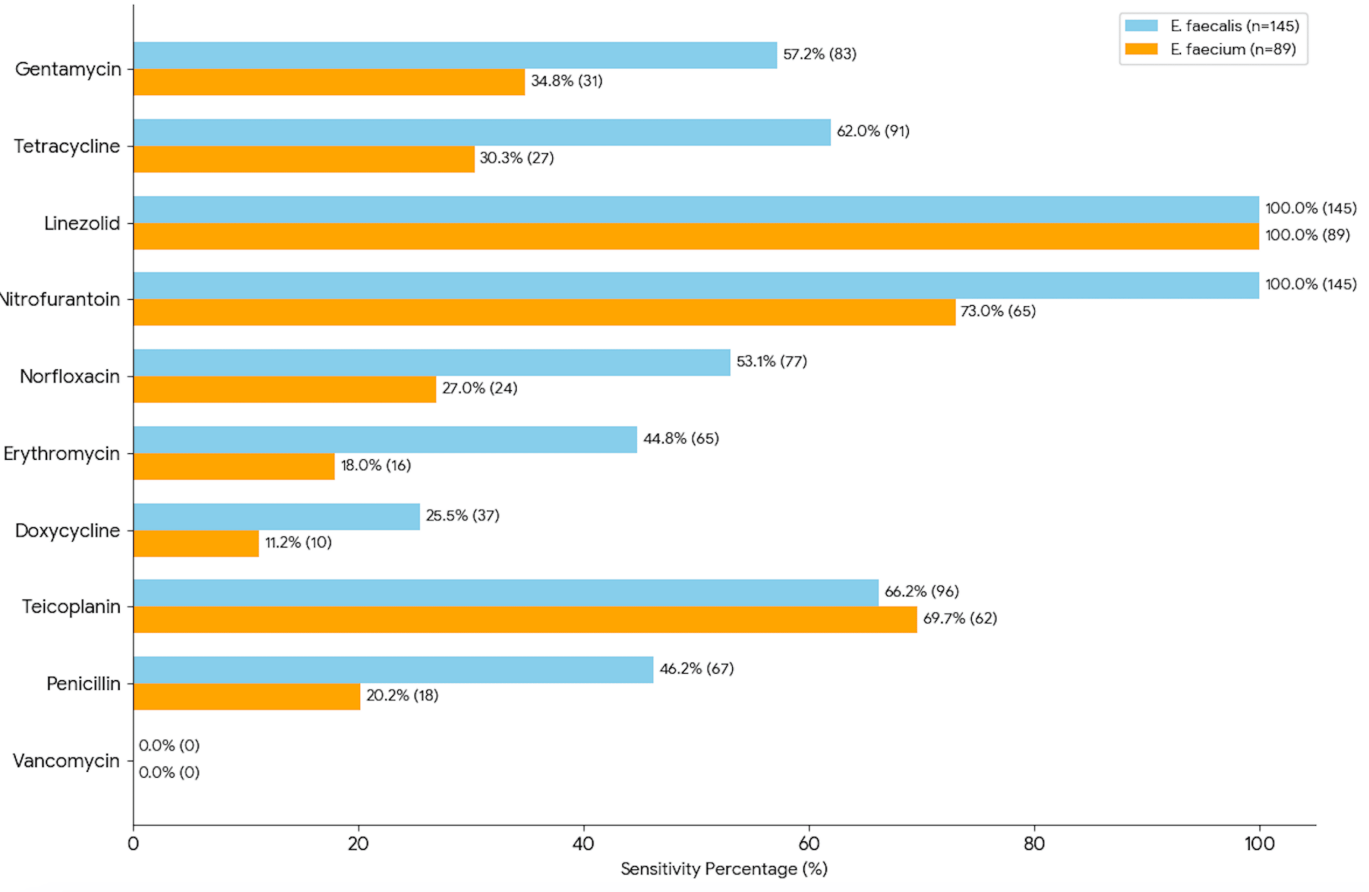
Antibiotic susceptibility profile of vancomycin-resistant enterococci *E. faecalis*: *Enterococcus faecalis*; *E. faecium*: *Enterococcus faecium*

All 234 VRE isolates demonstrated 100% resistance to vancomycin; linezolid was 100% effective, with all isolates being susceptible. *E. faecium* exhibited significantly higher resistance than *E. faecalis* to the following antibiotics: Penicillin (p = 0.0001), doxycycline (p = 0.013), erythromycin, norfloxacin, tetracycline, and gentamycin (p < 0.01). Teicoplanin resistance between the two species was not statistically significant(p= 0.686) as shown in Figure 2. 

Epsilometer strip minimum inhibitory concentration (E-strip MIC) testing was performed on 60 representative VRE isolates, revealing complete resistance to vancomycin, with all isolates exhibiting a MIC ≥ 32 μg/mL. Linezolid remained fully effective, with 59/60 (98.3%) isolates showing susceptibility and only one isolate displaying intermediate susceptibility. Daptomycin demonstrated high activity, with 58/60 (96.7%) isolates susceptible and two isolates showing intermediate resistance.

A subset of 20 VRE isolates was subjected to molecular characterization. All 20 isolates tested positive for the vanA gene, confirming high-level vancomycin resistance, while none were positive for the vanB gene, indicating that resistance in this study population was solely mediated by vanA. All samples also showed positive signals for the *Enterococcus *genus-specific 16S rRNA and internal controls, validating the integrity of the PCR reactions.

## Discussion

VRE has emerged as a critical nosocomial pathogen, particularly in tertiary care settings, due to its ability to cause invasive infections and exhibit resistance to multiple antimicrobial agents. The present study aimed to investigate the prevalence, species distribution, antimicrobial resistance profiles, and molecular characteristics of VRE isolates in a tertiary care hospital in North India, contributing to the growing body of national surveillance data.

The prevalence of VRE in our study was found to be 7.36%, aligning with previous reports from various regions of India. Similar prevalence rates have been reported by Kaarthiga et al. and Banerjee et al. in their study, both highlighting the significant burden of VRE in urban tertiary care centers [11,12]. In contrast, lower prevalence rates were noted by Taneja et al. and Modi et al., suggesting variability based on institutional infection control practices, patient demographics, and antimicrobial stewardship policies [13,14]. The high isolation rates from critical care areas such as ICUs and post-surgical wards further support the hypothesis that prolonged hospitalization, invasive procedures, and broad-spectrum antibiotic use are major contributors to VRE colonization and infection [15]. Our data confirm that VRE is most frequently isolated from urinary tract infections (7.8%), consistent with findings from Bhardwaj et al., who reported the urinary tract as a major site of VRE isolation in hospital settings [16].

*E. faecalis* was the predominant species in the present study, accounting for 61.7% of all *Enterococcus *isolates, while *E. faecium* comprised 38.3%. Among the 234 VRE isolates, *E. faecalis* and* E. faecium* accounted for 145 and 89 isolates, respectively. When analyzed at the species level, the rate of vancomycin resistance was comparable between *E. faecalis* (7.4%) and *E. faecium* (7.3%), indicating a similar resistance burden across both species despite differences in overall prevalence. Previous studies by Bhardwaj et al., Purohit et al., and Sivaradjy et al. have reported variable species distributions, with *E. faecalis* remaining the predominant isolate overall, while *E. faecium* is often overrepresented among resistant strains and isolates from intensive care settings [16-18]. Although *E. faecium* did not predominate among VRE isolates in the present study, its well-documented reduced susceptibility to beta (β)-lactams, aminoglycosides, and glycopeptides, along with its enhanced ability to survive in hospital environments, remains clinically relevant. Consequently, *E. faecium* has been classified by the World Health Organization as a high-priority pathogen for antimicrobial research and development. Its association with bloodstream and ICU infections underscores the importance of routine species-level identification in clinical laboratories to inform targeted therapy and infection control strategies [19].

The antibiotic susceptibility profiles in our study reveal high-level resistance among both *E. faecalis* and *E. faecium* isolates; penicillin resistance was markedly higher in *E. faecium* (79.77%) compared to *E. faecalis* (53.79%), corroborating previous literature that highlights the intrinsic and acquired resistance mechanisms in *E. faecium*, such as altered penicillin-binding proteins and efflux pumps. Similar resistance profiles have been reported by Bhardwaj et al. and Priyanka et al., indicating a consistent nationwide trend of multidrug resistance among *E. faecium* isolates [16,20]. High rates of resistance to doxycycline and erythromycin further limit oral therapeutic options, especially in outpatient and long-term care settings. In the present study, linezolid demonstrated high in vitro activity, with 100% susceptibility across both *E. faecalis* and *E. faecium*. However, these findings should be interpreted with caution, given the cross-sectional study design and the absence of MIC-based analysis. Additionally, sporadic reports of emerging linezolid resistance underscore the need for ongoing antimicrobial surveillance and robust stewardship practices to preserve the clinical utility of these agents, particularly for the management of severe VRE infections [17]. Nitrofurantoin showed 100% susceptibility among *E. faecalis* isolates from UTIs, whereas reduced susceptibility was observed in *E. faecium* (73.03%), highlighting the importance of routine species-level identification, judicious antibiotic selection, and continuous local susceptibility monitoring.

Molecular analysis of 20 randomly selected VRE isolates revealed 100% positivity for the vanA gene, with no detection of vanB, suggesting high gene expression and resistance burden. The predominance of vanA is consistent with earlier reports from Kashmir and North India, where vanA was identified in >90% of VRE isolates [5,21]. However, a study from Mumbai has indicated an increasing emergence of vanB (15%), signaling a potential shift in resistance patterns, particularly in Western India [22]. The exclusive presence of vanA in our setting suggests clonal dissemination and potential horizontal gene transfer, commonly associated with vanA, within the hospital environment. Continuous molecular surveillance is vital to detect emerging vanB or vanD variants, which may influence therapeutic decision-making due to differing resistance phenotypes.

Limitations

The study has several limitations that should be acknowledged. Being conducted at a single tertiary care hospital (Dr. RMLIMS, Lucknow), the findings may have limited generalizability to other healthcare settings, particularly rural hospitals or primary care institutions. The analysis lacked detailed patient-level risk factor assessment and outcome data, which restricts the evaluation of clinical impact and limits extrapolation to patient management and prognosis. Molecular characterization was performed on only 20 VRE isolates using multiplex PCR targeting vanA and vanB genes, which may not fully capture the genetic diversity of vancomycin resistance mechanisms prevalent in the region. Furthermore, advanced genotypic typing and clonal analysis methods such as pulsed-field gel electrophoresis, multilocus sequence typing, or whole-genome sequencing were not undertaken, limiting insights into clonal relatedness, transmission dynamics, and outbreak potential of VRE strains.

## Conclusions

This study demonstrates a 7.36% prevalence of VRE in a North Indian tertiary care hospital, with UTIs and BSIs being the most commonly affected clinical syndromes. *E. faecium* exhibited higher levels of multidrug resistance compared to *E. faecalis*, while linezolid retained good in vitro activity against VRE isolates. Molecular analysis identified vanA as the predominant mechanism mediating vancomycin resistance among the isolates studied. Although the exclusive detection of vanA suggests a common resistance mechanism, definitive conclusions regarding clonal dissemination cannot be drawn in the absence of genotypic or clonal typing. Nevertheless, these findings highlight the importance of routine species-level identification, judicious antimicrobial stewardship, and ongoing molecular surveillance to monitor resistance patterns and limit the spread of VRE in hospital settings.
